# Characterization of Corning EPMA Standard Glasses 95IRV, 95IRW, and 95IRX

**DOI:** 10.6028/jres.107.057

**Published:** 2002-12-01

**Authors:** Paul Carpenter, Dale Counce, Emily Kluk, Carol Nabelek

**Affiliations:** Alliance for Microgravity Materials Science and Applications, SD46/USRA/NASA, Marshall Space Flight Center, AL 35812; Los Alamos National Laboratory, EES-1 MS D469, Los Alamos, NM 87545; Department of Geological Sciences, University of Missouri, Columbia, MO 65211

**Keywords:** Corning, epma, glass, homogeneity, microprobe, standard, trace element, eds, 95IRV, 95IRW, 95IRX

## Abstract

The preparation, synthesis, and characterization of Corning trace-element glasses 95IRV, 95IRW, and 95IRX by bulk chemical and electron microprobe techniques is discussed. Working values for the doped elements in the 95-series glasses are established. Blank values have been determined by both bulk chemical and electron microprobe analysis, and important x-ray interferences are highlighted. Chemical homogeneity both within a rod cross-section, and along cane length has been documented. These glasses are standard reference materials intended for use as both primary and secondary electron microprobe standards.

## 1. Introduction

In 1971, Art Chodos and Arden Albee, of the Division of Earth and Planetary Sciences at Caltech, contracted Corning Glass Works^1^ to produce synthetic glasses containing a number of elements at approximately 0.01 mass fraction concentration, with the intention of using these glasses as trace element reference standards for electron-probe microanalysis (EPMA). The master list of elements was divided into three groups in order to avoid x-ray peak overlaps within a given glass standard. Three glasses were subsequently produced, 95IRV, 95IRW, and 95IRX (informally known in the microanalysis community as Corning/Caltech glasses GLV, GLW, and GLX, respectively), which were doped with the following elements in a Ca-Mg-Al borosilicate glass matrix. Glass 95IRV contains K, Ti, Cr, Fe, Ce, and Hf, and is green in color. Glass 95IRW contains V, Mn, Co, Cu, Cs, Ba, La, and Th, and is blue in color. Glass 95IRX contains Ni, Zn, Rb, Sr, Y, Zr, Pb, and U, and is brown in color. The concentration of these elements is sufficient to use the standards for primary calibration by EPMA, and the glasses have since been used for the analysis of a wide range of materials. This paper describes the synthesis, chemical characterization by wet chemical and x-ray techniques, and chemical homogeneity measurements performed on these EPMA standard reference materials. Information concerning the preparation and early characterization of these standards is based on correspondence and documents organized by Art Chodos, and subsequently assumed by Carpenter who has briefly summarized this early work [1].

## 2. Preparation of Corning 95IRV, 95IRW, and 95IRX Glasses

Corning Glass Works had previously produced the SRM 612, 614, and 616 trace element glasses for NIST (then National Bureau of Standards). These glasses were composed of a Na-Al silicate matrix, were doped with a maximum level of 5.0 × 10^−4^ of each element, and were batched in comparatively large 68 kg lots. In contrast, the matrix composition of the 95-series glasses was carefully chosen to exclude alkali elements (i.e., Na) in major concentration, in order to avoid the problem of alkali migration under the electron beam for application as EPMA reference standards. The 95-series glasses were also doped with a higher level of each element, and each glass was batched in a 0.91 kg lot and delivered at the bargain price of $200 per glass. The source materials for each oxide dopant were selected by Corning personnel from reagents consistent with production of other research-grade synthetic glasses, and approximately 1.36 kg of starting material were used to generate each glass. The materials used and the weighed-in quantities of each reagent are listed in Table 1. An error was apparently made in the calculation of the amount of reagent needed to produce a target concentration of 0.01 massfraction and as a result, in the process of batching of the glasses, the amounts of each oxide were nominally 0.0079 mass fraction rather than the intended 0.01 mass fraction (there was apparently no error in the weighing process). Because these glasses contain substantial quantities of the dopant elements, and therefore associated oxygen would be appropriate in a chemical analysis, it was decided early on to report the concentrations as oxide rather than as the element. The weighed-in concentrations of all elements are reported as the most commonly used oxide for purposes of reporting analyses (rather than the oxide used as source material), and for this reason the quantities of V, Mn, Fe, Ce, and U are different from the nominal value of 0.0079 mass fraction. Notice also that K and Cr were batched using K_2_CO_3_ and K_2_C_2_O_7_, and one would expect a correlation to exist between Cr and K in 95IRV for this reason. No information is currently available concerning the oxidation state of elements in the 95-series glasses.

After batching, each powder was subjected to two cycles of melting and stirring in a Pt-Rh lined container in a furnace. After these homogenizing steps, a 0.65 cm diameter glass cane was drawn from the melt and was subsequently cut into 9 rods, each approximately 13 cm long, which were then numbered for purposes of cataloging and tracking. Distribution of the glasses to end-users for use as EPMA standards was in the form of 0.65 cm diameter disks cut from the end (i.e., first drawn) of each cane. Qualitative wavelength-dispersive x-ray fluorescence (WDXRF) x-ray scans were provided by Corning to document the presence of the requested trace elements in each glass (and interestingly, also documented the existence of Sr contamination in glasses 95IRX and 95IRW). The weighed-in quantities were initially used as working values for the trace elements, until further analytical work to characterize the actual concentrations of the dopants was performed. These weighed-in values provide a check on the final glass composition as well as subsequent analytical measurements, but also can be used to identify loss of material during the glass-forming process.

## 3. Bulk Chemical Analysis

Bulk chemical analysis has been performed entirely by volunteers from the geological and analytical chemistry communities, and has been documented in progress reports by Carpenter [1], and Carpenter, Counce, Kluk, and Nabelek [2]. The existing bulk chemical analyses of glasses 95IRV, 95IRW, and 95IRX are summarized in Tables 2, 3, and 4, respectively. Analytical work began in the 1970s, and was first performed at the Oregon State University Radiation Center using instrumental neutron activation analysis (INAA). This work was performed by an undergraduate student for a research project, under the supervision of Dr. Roman Schmitt. Because thermal neutrons are strongly absorbed by boron, accuracy of the INAA results was degraded, and due to other problems these results are not presented here. However, this data set was critical in alerting all to the previously mentioned error in batch calculation.

Eugene Jarosewich of the Smithsonian Institution performed analyses by colorimetry and flame emission on two samples of material from the beginning of each cane. Flame emission was used to measure K in 95IRV, and colorimetry was used to measure Ti, Cr, and Fe in 95IRV, Mn and Co in 95IRW, and Ni and Zn in 95IRX. (Colorimetry was presumably also used to determine Mg, Al, Si, and Ca in 95IRV). Analytical accuracy in these runs was determined by analyzing known rock and metal standards as unknowns. The secondary rock standards used were BRC-1 for K and Mn, G-2 for Ti and Fe, PCC-1 for Cr and Ni, PTS-1 for Cr, and G-1 for Zn (using method of additions). The secondary steel standards used were Steel 8i for Mn, and Steel 126a for Co. Jarosewich achieved excellent accuracy relative to these secondary standards. J. L. Elize of Corning used gravimetric analysis to determine B in glasses 95IRV and 95IRW, and Zr in glass 95IRX. He also used flame emission to determine K in 95IRV, Cs in 95IRW, and Rb in 95IRX.

Material from the beginning, middle, and end of each cane was given to Jun Ito at the University of Chicago, who performed atomic absorption spectrophotometry (AA) in order to characterize the glasses and to evaluate homogeneity along the cane length. These analyses are the only documentation, to date, of homogeneity along the length of the drawn cane, which would reflect any changes in melt chemistry during the glass drawing process. The glass samples were finely divided in corundum mortars, then dissolved in nitric acid, and 100 mL dilute solutions were prepared for AA analysis. The analyses were calibrated by standard solutions and secondary standards were also analyzed as unknowns. Uncertainties of these measurements were estimated by Ito to be 1.0 × 10^4^ mass fraction of the oxide.

More recently, Carpenter, Counce, Kluk, and Nabelek [2] initiated a second round of analytical work using the techniques of inductively coupled plasma atomic-emission spectrometry (ICP-AES), x-ray fluorescence spectrometry (XRF), and EPMA. The intention of this second round was to further refine the concentrations of doped elements, determine what blank values and cross-contamination exists among the glasses, and measure the chemical homogeneity of the glasses.

Bulk analysis using ICP-AES was performed by Dale Counce at Los Alamos National Laboratory (LANL), and by Carol Nabelek at the University of Missouri. These results are listed in Tables 2, 3, and 4 for glasses 95IRV, 95IRW, and 95IRX, respectively. Approximately 5 g of each glass was used for duplicate analyses by ICP-AES. Samples were ground, dried, and prepared by both microwave acid digestion and fusion methods. Material prepared by microwave acid digestion used approximately 0.25 g sample, 3.5 mL HCl, 2 mL HNO_3_, and 1.5 mL HF, and was heated to a pressure of 1378 kPa and held for 30 min in digestion bombs. Material prepared by fusion used approximately 0.25 g sample with 2 g LiBO_2_ flux, and was heated at 950 °C for 30 minutes followed by dissolution of the fused bead in 5 % HNO_3_. While the microwave acid digestion method is typically used for volatile and trace metals, the use of HF is necessary to dissolve silicate material, and may result in precipitation of insoluble fluorides. Substantially low concentrations were observed for several elements in material processed using the microwave acid digestion method (i.e., Ti, Sr, Y, Ba, La, Ce, and Th), apparently representing extraction problems. However, the fusion method worked well for these elements as well as Hf and U. Good extractions were obtained using the microwave acid digestion technique for K, V, Cr, Mn, Fe, Co, Ni, Cu, Zn, Pb, and U. Both Zr and Hf behave in a chemically similar manner, and lower concentrations are observed for the samples processed with the microwave acid digestion technique compared to the fusion method. Due to the paucity of data for these elements, both sets of data have been reported, but it does appear that the fusion results are superior. These comments pertain to the doped levels of the elements in the 95-series glasses (i.e., approximately 0.0079 mass fraction), but for blank levels good values were apparently obtained using the microwave acid digestion method where that approach did not work for the higher concentrations. That is, both the acid digestion and fusion techniques yield identical results at low concentration. These issues concerning sample preparation and dissolution techniques warrant further work. In particular, accurate analysis for Ce in 95IRW and 95IRX was not possible apparently due to sample preparation problems.

Bulk analysis using wavelength dispersive XRF was performed by Emily Kluk at Los Alamos National Laboratory (LANL). These results are listed in Tables 2, 3, and 4 for glasses 95IRV, 95IRW, and 95IRX, respectively. Approximately 5 g of sample was first crushed with a mortar and pestle, then pulverized in an alumina ceramic shatterbox. Sample splits from this powder were dried at 100 °C for 4 hours, then allowed to equilibrate at ambient temperature for 12 hours. Two fusion disks were made in graphite crucibles using LiBO_2_ flux at 9:1 and 36:1 dilutions, using 9 g of lithium tetraborate and heating for 1 hour at 1100 °C in a muffle furnace. Additional splits were heated at 1000 °C in order to obtain loss on ignition measurements. Measurements were made on an automated Rigaku wavelength-dispersive XRF system, using procedures appropriate for trace element analysis of silicate rock samples that have been established in the LANL laboratory. Element compositions were calculated by comparing the measured x-ray intensities to those of 21 rock standards for the 9:1 dilution, and to 23 rock standards for the 36:1 dilution. The “consensus” values of Govindaraju [3] were used for the calibration standards. Final values were obtained by averaging measurements, where appropriate, for the 9:1 and 36:1 dilutions. Problems were encountered with analysis of Ni and Zn (in 95IRX only), possibly due to reduced solubility of these elements in the flux. The possibility of contamination of V due to contaminated crucibles was discounted by analyzing quartz sand, and a secondary standard was processed with the 95-series glasses in order to detect sample preparation problems as part of a long term monitoring project. Several x-ray peak interferences were encountered during these runs. In 95IRV, both the CrKβ and CeLγ lines interfere with MnKα. In 95IRW, the ThLβ2 line interferes with ZrKα, and the VKβ line interferes with CrKα. In 95IRX, the UMβ line interferes with KKα, and the PbLγ1 line interferes with YKα. Values for these interfered elements are therefore not reported. Because the 95-series glasses contain relatively large concentrations of trace elements compared to the rock standards that were used, several elements were out of the calibration range. However, in comparing the XRF data to those from other techniques, the agreement is excellent. XRF analytical data have also been obtained from Johnson Space Center, where the glasses were analyzed by an unknown individual; these results are also included for comparison with the data acquired at LANL.

## 4. EPMA Studies

The technique of EPMA has been utilized by Carpenter, at both Caltech and Marshall Space Flight Center (MSFC), to study several aspects of micro-analysis that are important to the use of the Corning glasses as standard reference materials. EPMA studies have been performed on the glasses using the JEOL JXA-733 electron microprobe at Caltech, equipped with five wavelength- dispersive spectrometers (WDS), and on the JEOL 8900/R electron microprobe at MSFC, equipped with four WDS. Both instruments have energy dispersive spectrometers (EDS) and customized software that has been used for quantitative analysis and homogeneity measurements. Qualitative analysis has been performed on the microprobe using both WDS and EDS. Carpenter has also used energy dispersive XRF at Caltech to confirm the presence of dopant elements, evaluate peak overlaps and other artifacts that are specific to EDS, and to screen the 95- series glasses for contaminant elements. A Kevex 0700 EDS x-ray analyzer using a Rh x-ray tube and a secondary x-ray target carousel was used for this purpose. These EDSXRF spectra have been used in conjunction with qualitative wavelength scans obtained on the electron microprobe to verify the existence of low intensity x-ray peaks. The microprobe WDS has been used to document the characteristic x-ray peak position, any peak overlaps that may exist, and any other artifacts such as absorption edges or multiple-order peak reflections. Wavelength scans were obtained on the 95-series glasses typically at 25 kV accelerating potential, 250 nA probe current, and a beam diameter of 100 μm. Numerous runs at different acquisition times were employed and wavelength scans were obtained using TAP, LIF, and PET analyzing crystals and both gas-flow and sealed x-ray detectors where appropriate. Wavelength scans from the three glasses were superimposed to compare peak size, position, and possible overlaps. In this way it was possible to select a master set of background offset positions that would be free of interferences for all three glasses. These WDS wavelength scans also serve to visually confirm the extent of cross-contamination of elements in all three glasses.

## 5. Bulk Chemistry Homogeneity Data

The homogeneity of the 95-series glasses has been documented using both bulk chemical and EPMA. As discussed previously, Ito analyzed samples taken from the beginning, middle, and end of each glass cane. For 95IRV these are analyses V3a, V3b, and V3c (Table 2), for glass 95IRW these are analyses W3a, W3b, and W3c (Table 3), and for 95IRX these are X3a, X3b, and X3c (Table 4). For the elements K, Ti, Cr, and Fe in glass 95IRV, V, Mn, Co, Cu, and Ba in glass 95IRW, and Zn and Sr in 95IRX, the variation in concentration along cane length is less than or equal to the analytical uncertainty 0.0001 mass fraction of the oxide as quoted by Ito, and therefore appear to be homogeneously distributed along the length of the canes. The variations of Cs in glass 95IRW, and of Ni and Rb in glass 95IRX are somewhat greater, and appear to decrease in concentration with distance along the cane. The concentration of Pb in 95IRX appears to decrease, then increase again. However, it should be noted that the total variation in these elements is less than the standard deviation of analyses obtained by the different analysts and techniques on material from the beginning of each cane. Based on this information, it appears that the 95-series glasses are homogeneous with respect to cane length.

## 6. EPMA Homogeneity Data

In order to evaluate the homogeneity on a micron scale, and to determine if any radial variations in homogeneity exist, measurements have been made by EPMA on discs cut perpendicular to the length of the cane. These discs were mounted, micropolished, and coated with evaporated carbon as per normal sample preparation for EPMA. These discs have been carefully inspected using secondary electron and backscattered electron imaging, and no inclusions or other features were observed by Carpenter or reported by any users. The homogeneity of the standard glasses has been measured on these polished discs using both linear traverses that sample the diameter of a disc, as well as a point-count grid pattern that effectively samples the entire disc cross-section. For these EPMA measurements the average working values for each oxide in the 95-series glasses were used to establish the glass composition, and a self-standardization was performed using all three glasses. That is, 95IRV was used to standardize K, Ti, Cr, Fe, Ce, and Hf, and homogeneity measurements were then made on 95IRV relative to this self-standardization. This process was repeated for 95IRW and 95IRX and the suite of elements in those glasses.

Analytical conditions were carefully chosen to obtain high x-ray count rates requiring a relatively high probe current, but to avoid beam damage to the sample, and for the purposes of homogeneity measurements, to sample as large a volume as possible while maintaining x-ray focus with the WDS. The reader should be cautioned in this respect that a finely focused electron beam at high probe current should not be used on these glasses, as this will cause migration of selected elements to occur, as well as possible burning of the sample. Time-dependent counting experiments were conducted on a fixed sample spot in order to demonstrate the time invariant x-ray intensity at an accelerating potential of 20 kV, a probe current of 100 nA, and beam diameters of approximately 1 μm (i.e., a focused beam), 5 μm, 10 μm, 20 μm, 50 μm, and 100 μm. Measurements were performed on KKα in glass 95IRV, CsLα in glass 95IRW, and RbLα in glass 95IRX, since one would expect these alkali elements to migrate most readily under electron irradiation. For each counting experiment, the WDS was carefully tuned to the x-ray peak, then a fresh analytical spot was selected. The WDS was left at the peak position for the duration of the experiment and no background measurements were obtained, since only relative x-ray intensity changes were being monitored. Each experiment acquired x-ray counts for the selected element at 20 s intervals, for a total count time of 30 min. The results of these experiments indicate that K migrates most readily and exhibits an immediate decrease in x-ray intensity at a probe diameter of 5 μm, but at a diameter of 10 μm a decrease in intensity was not observed until approximately 1000 s had elapsed. No decrease in x-ray intensity was observed for KKα at probe diameters of 20 μm, 50 μm, and 100 μm over the duration of the 30 min experiment. For CsLα in 95IRW and RbLα in 95IRX, an immediate decrease in x-ray intensity was observed only for a focused electron beam. No decrease in x-ray intensity was observed for CsLα in 95IRW at 5 μm, 10 μm, 20 μm, 50 μm, or 100 μm probe diameter over the duration of the same time period. A less clearly defined decrease in x-ray intensity for RbLα in 95IRX occurred after several hundred seconds at a probe diameter of 5 μm, but no decrease was observed for diameters of 10 μm, 20 μm, 50 μm, and 100 μm. In these measurements, the count rate was highest for KKα, intermediate for CsLα, and low for RbLα, and as a result the precision of these measurements was also highest for K, and lowest for Rb. From these experiments it is clear that one should use a probe diameter no less than 20 μm at a probe current of 100 nA, and it would be prudent to use a larger diameter if at all possible.

For the purposes of homogeneity measurements, counts were obtained at 25 kV accelerating potential, 250 nA probe current, 100 μm beam diameter, and count times of 250 s to 375 s per element at each point in a 60 point grid covering the entire disc. Again, the time-invariant x-ray intensity was confirmed using the previously discussed strategy at these analytical conditions. Wherever possible, elements were measured using the analyzing crystal with the highest resolution, so that the numerous x-ray interferences observed in the master list of elements in the 95-series glasses could be minimized or avoided altogether. The microprobe at MSFC is equipped with a JEOL H-type WDS that obtains a higher count rate at the expense of peak resolution, compared to a normal WDS. This spectrometer was used for elements with lower intensities where interferences were not important, and in fact was essential for their measurement. The Kα x-ray line was used to analyze elements K, Ti, V, Cr, Mn, Fe, Co, Ni, Cu, and Zn; the Lα x-ray line was used for Rb, Sr, Y, Zr, La, Ce, Hf, and Pb; and the Mα x-ray line was used for U and Th. The PET analyzing crystal was used to measure K, Ti, Rb, Sr, Y, Zr, Cs, Th, and U (and the H-type spectrometer was used for Rb, Sr, and Y). The LiF analyzing crystal was used to measure V, Cr, Mn, Fe, Co, Ni, Cu, Zn, Ba, La, Ce, Hf, and Pb (and the H-type spectrometer was used for Cr, V, Ba, La, and Ce).

Several homogeneity measurements were performed over a period of about 1 year. These measurements were used to calculate a sigma ratio *σ* for each element in each glass, which is the observed standard deviation of counts acquired on all points divided by the square root of the mean of the counts (i.e., the standard deviation expected from counting statistics alone). These *σ* values are tabulated in columns V_s_, V_w_, and V_x_ of Tables 5, 6, and 7 for the 95-series glasses, and range from a low of 2.1 for Mn to higher values of 5.2 for Rb and 6.5 for Cs. Note that while a *σ* value of 1 indicates a homogeneous material, the concentration of these elements is lower by a factor of 10 than those for which these measurements are normally made and much longer counting times are required to obtain a given level of counting statistics. Instrumental stability becomes critically important in these measurements, and the lowest *σ* values are reported here for a given element, as several runs exhibited probe current instability that produced an artificially high *σ*. The same data that were used to calculate the *σ* values were also used to generate contour plots of element concentration as a function of position on the sample disc (these can be obtained from Carpenter upon request). These plots, coupled with the homogeneity measurements lead to the following conclusions. Firstly, there are no obvious variations in concentration with radial distance in a sample disc, nor are any hot spots or depressions in concentration observed from the measurements. Secondly, although it appears that Cu and Cs in 95IRW, and Rb in 95IRX were preferentially lost from the glasses during melting, as is evident in comparing the weighed-in values with the average bulk chemical data, these elements do not exhibit any radial variation in concentration. The bulk chemistry data of Ito do not suggest homogeneity problems for Cs and Rb, but the EPMA measurements do indicate that these elements are less homogeneously distributed than the other dopants.

## 7. EPMA Measurement of Cross-Contamination and Blank Values

EPMA measurements were made to determine the extent of cross-contamination, to evaluate blank levels of non-dopant elements, and to measure the apparent concentration due to peak interferences on the measured elements. A self-standardization procedure was again employed, in which each 95-series glass was used for calibration of the doped elements, using the average-value for the oxide concentration from Tables 5, 6, and 7. Then the other two glasses were analyzed as unknowns relative to this standardization. For example, 95IRV was used to standardize for K, Ti, Cr, Fe, Ce, and Hf, using background offsets that would be appropriate for all three glasses; 95IRW and 95IRX were then analyzed as unknowns for those elements. This procedure was then repeated for the other standards, where the element suite in 95IRW was analyzed in 95IRV and 95IRX relative to the 95IRW calibration, and finally the 95IRX suite was analyzed in 95IRV and 95IRW relative to the 95IRX calibration.

## 8. Discussion

Summaries of the bulk chemistry data for glasses 95IRV, 95IRW, and 95IRX are listed in Tables 5, 6, and 7, respectively. These tables contain the average values (Vba, Wba, and Xba) and standard deviation (Vbsd, Wbsd, and Xbsd) of the bulk chemistry data, and include both doped and apparent blank values for the master suite of elements. We recommend the use of the average bulk chemistry data for the 95-series glasses as EPMA standards. The average blank values (Vpa, Wpa, and Xpa) and their standard deviations (Vpsd, Wpsd, and Xpsd), as determined by EPMA using the internal 95-series calibration, are listed for comparison. It should be stressed that these are not corrected for x-ray peak interference, so that the magnitude of the interference may be demonstrated, and of course one would not expect good agreement with the bulk chemistry data in the presence of an x-ray interference. Columns Vpi, Wpi, and Xpi denote the presence of a peak observed on a WDS scan, as well as any x-ray interferences, which may be at the peak of interest, or close by to cause problems with background measurement. The columns Vw-ba, Ww-ba, and Xw-ba compare the average bulk chemistry data with the weighed- in values, on a percentage basis. One goal of this study is to establish working values for the 95- series glasses and to evaluate the accuracy of the chemistry data set. One assessment of the accuracy of the bulk chemistry data is to rank the elements in order of increasing standard deviation of the bulk chemistry data (i.e., columns Vbsd, Wbsd, and Xbsd of Tables 5, 6, and 7). This presumably ranks the element with best agreement amongst the different analysts and techniques as being most accurately analyzed. While the weighed-in values were initially used for the glasses, there is apparently no relation between this agreement and the difference between weighed-in and analyzed values, so it is not clear how to interpret adherence to weighed-in values in assessing the analytical accuracy.

### 8.1 Glass 95IRV

Glass 95IRV is nominally doped with K, Ti, Cr, Fe, Ce, and Hf. For 95IRV the element ranking in order of increasing standard deviation is Cr (2.3 × 10^−4^ mass fraction), Fe (3.9 × 10^−4^), Ti (4.0 × 10^−4^), Hf (5.5 × 10^−4^), K (7.3 × 10^−4^), and Ce (7.6 × 10^−4^). Thus, it seems likely that the Cr data for 95IRV are the most accurate as there is good agreement between the techniques, and the Ce data are comparatively the least accurate. There are only two analyses for Ce, and problems have been observed due to sample digestion with Ce in general. Note that for Hf there is disagreement between ICP-AES data for acid digestion vs. fusion techniques. Compared to 95IRW and 95IRX, the bulk chemistry data for 95IRV shows the poorest agreement. As determined by bulk chemical techniques, glass 95IRV has blank levels of less than 5.0 × 10^−5^ mass fraction as oxide for elements V, Co, Cu, La, Zn, and P, a blank level of less than 1.0 × 10^−4^ mass fraction for elements Mn, Y, and Pb, and a blank level of less than 2.0 × 10^−4^ mass fraction for elements Th, Ba, and Na (the listed elements are in order of increasing concentration). The highest contaminant is Sr, with a concentration of 8.0 × 10^−4^ mass fraction. Excellent agreement is observed for the blank values determined by bulk chemistry and EPMA techniques, with the exception of Sr, for which there is at this time no explanation of the discrepancy. Sigma ratio values derived from homogeneity measurements using EPMA are also listed in Table 5. These *σ* ratios indicate that Cr, Fe, and Ce are apparently more homogeneously distributed than K, Ti, and Hf. Contour maps were produced using the point count data from the homogeneity measurements, and these maps exhibit the following ranges in oxide concentration (in mass fraction) for 95IRV: K (3.0 × 10^−4^), Ti (2.0 × 10^−4^), Cr (2.5 × 10^−4^), Fe (3.0 × 10^−4^), Ce (4.0 × 10^−4^), and Hf (2.0 × 10^−4^). That is, of 60 analyses in a point count grid that spans the cane diameter, each doped element in 95IRV was typically observed to have a total range of 2.0 × 10^−4^ mass fraction to 4.0 × 10^−4^ mass fraction oxide concentration, relative to the average bulk chemistry value. For example, for HfO_2_ the oxide concentration is (0.00744 ± 0.0001) mass fraction, and for K_2_O the value is (0.00792 ± 0.00015) mass fraction. These results indicate that the glass homogeneity is much better than the disagreement associated with bulk analysis by the different techniques.

### 8.2 Glass 95IRW

Glass 95IRW is nominally doped with V, Mn, Co, Cu, Cs, Ba, La, and Th. For 95IRW the element ranking in order of increasing standard deviation is Th (one point only), La (0.4 × 10^−4^ mass fraction), V (1.1 × 10^−4^), Cs (1.2 × 10^−4^), Ba (1.2 × 10^−4^), Mn (1.8 × 10^−4^), Co(1.8 × 10^−4^), and Cu (1.9 × 10^−4^). Thus, it seems likely that the La data for 95IRW are the most accurate, and the Cu data are the least accurate. Note that for Th there currently exists only one analysis, ICP-AES using the fusion technique. There is an indication that Cu and Cs may have been lost during the glass forming process, as the average bulk analyses are about 10 % lower than the weighed-in values. Compared to 95IRV and 95IRX, the bulk chemistry data for 95IRW shows the best agreement among the techniques and analysts. As determined by bulk chemical techniques, glass 95IRW has blank levels of less than 5.0 × 10^−5^ mass fraction as oxide for elements U, Cr, Na, and Hf, a blank level of less than 1.0 × 10^−4^ mass fraction for elements P, Ni, Pb, Zr, Ti, Zn, and Y, and a blank level of less than 2.0 × 10^−4^ mass fraction for K (the listed elements are in order of increasing concentration). Glass 95IRW contains both Fe and Sr at a concentration of 4.4 × 10^−4^ mass fraction and 8.4 × 10^−4^ mass fraction, respectively. Excellent agreement is observed for the blank values determined by bulk chemistry and EPMA techniques, with the exception of Fe and Sr, for which there is also at this time no explanation for the discrepancy. It is very important to note that 95IRW has a built-in interference on the primary LaLα1 analytical line, namely the CsLβ1, Lβ3, and Lβ4 peaks. It is imperative to use the LiF analyzing crystal to resolve this interference. Sigma ratios (*σ*) derived from homogeneity measurements using EPMA are also listed in Table 6. These *σ* ratios indicate that Mn, Th, and Co are apparently more homogeneously distributed than La, Cu, V, and Ba. The homogeneity of Cs is apparently worse by a factor of 2. Contour maps were produced using the point count data from the homogeneity measurements, and these maps exhibit the following ranges in oxide concentration (in mass fraction) for 95IRW: V (2.5 × 10^−4^), Mn (2.0 × 10^−4^), Co (2.0 × 10^−4^), Cu (3.5 × 10^−4^), Cs (1 × 10^−3^ or 10 × 10^−4)^, Ba (2.0 × 10^−4^), La (2.0 × 10^−4^), and Th (3.5 × 10^−4^). That is, of 60 analyses in a point count grid that spans the cane diameter, each doped element in 95IRW was typically observed to have a total range of 2.0 × 10^−4^ mass fraction to 3.5 × 10^−4^ mass fraction oxide concentration, with the exception of Cs, which exhibited a range of about 1 × 10^−3^ mass fraction, relative to the average bulk chemistry value. For example, for MnO the oxide concentration is (0.00637 ± 0.00010) mass fraction, and for Cs_2_O the value is (0.00710 ± 0.0005) mass fraction. These results indicate that the glass homogeneity is comparable to the disagreement associated with bulk analysis by the different techniques, with the exception of Cs, for which the apparent homogeneity is comparatively poorer.

### 8.3 Glass 95IRX

Glass 95IRX is nominally doped with Ni, Zn, Rb, Sr, Y, Zr, Pb, and U. For 95IRX the element ranking in order of increasing standard deviation is Pb (0.9 × 10^−4^ mass fraction), U (1.0 × 10^−4^), Rb (1.6 × 10^−4^), Zn (1.9 × 10^−4^), Ni (7.3 × 10^−4^), Zr (3.2 × 10^−4^), Sr (3.4 × 10^−4^), and Y (4.9 × 10^−4^). Thus, it seems likely that the Pb data for 95IRX are the most accurate, and the Y data are the least accurate. Note that for Y there are at this time two analyses only. It seems from comparison of the weighed-in values with the average bulk chemistry data that 37 % of Rb was lost during glass production. Compared to 95IRV and 95IRW, the bulk chemistry data for 95IRX shows intermediate agreement. As determined by bulk chemical techniques, glass 95IRX has blank levels of less than 5.0 × 10^−5^ mass fraction as oxide for elements Cr, V, Co, and La, a blank level of less than 1.0 × 10^−4^ mass fraction for elements Mn, Cu, P, and Ti, and a blank level of less than 2.0 × 10^−4^ mass fraction for elements Th, Ba, and Hf (the listed elements are in order of increasing concentration). The concentrations of K and Fe have values of 2.8 × 10^−4^ mass fraction and 6.3 × 10^−4^ mass fraction, respectively. Generally excellent agreement is observed for the blank values determined by bulk chemistry and EPMA techniques, with the exception of Fe, for which again there is at this time no explanation for the discrepancy. Sigma ratios (*σ*) derived from homogeneity measurements using EPMA are also listed in Table 7. These *σ* ratios indicate that Y, Zr, U, and Sr are apparently more homogeneously distributed than Zn, Ni, Pb, and Rb. Contour maps were produced using the point count data from the homogeneity measurements, and these maps exhibit the following ranges in oxide concentration (in mass fraction) for 95IRX: Ni (4.0 × 10^−4^), Zn (3.5 × 10^−4^), Rb (4.0 × 10^−4^), Sr (2.5 × 10^−4^), Y (2.5 × 10^−4^), Zr (4.0 × 10^−4^), Pb (4.0 × 10^−4^), and U (3.0 × 10^−4^). That is, of 60 analyses in a point count grid that spans the cane diameter, each doped element in 95IRX was typically observed to have a total range of 2.5 × 10^−4^ mass fraction to 4.0 × 10^−4^ mass fraction oxide concentration, relative to the average bulk value. For example, for SrO the oxide concentration is (0.00762 ± 0.00013) mass fraction, and for Rb_2_O the value is (0.00494 ± 0.00020) mass fraction. These results indicate that the glass homogeneity is comparable to the disagreement associated with bulk analysis by the different techniques. These data also show that although Rb was lost during glass formation, it appears to be homogeneously distributed.

## 9. Distribution of Standard Glasses

The Corning standard glasses 95IRV, 95IRW, and 95IRX were developed for the Microbeam Analysis Society. Distribution of the standards is now being handled by the Smithsonian Institution. The material is distributed as a coarsely crushed glass, placed in a vial, or in disc format [4]. The Smithsonian has assigned USNM numbers to the glasses as follows: Glass 95IRV is USNM 117083, Glass 95IRW is USNM 117084 and Glass 95IRX is USNM 117085.

## 10. Conclusions

Bulk and microanalytical chemistry data for Corning EPMA glasses 95IRV, 95IRW, and 95IRX are reported and discussed. It is recommended that the average bulk chemistry data be adopted as the working values for each glass for use as EPMA standard reference material (i.e., Vba of Table 5 for 95IRV, Wba of Table 6 for 95IRW, and Xba of Table 7 for 95IRX). In general there is excellent agreement between both the bulk chemistry data and the self- standardized EPMA data, and the glasses appear in general to be homogeneous with respect to disc diameter and cane length.

## Figures and Tables

**Table 1:** Source materials and weighed-in quantities for glasses 95IRV, 95IRW, and 95IRX

Oxide	Source Material^a^	95IRV		95IRW		95IRX	
		Oxide g	Mass Fraction ×10^2^	Oxide g	Mass Fraction ×10^2^	Oxide g	Mass Fraction ×10^2^
B_2_O_3_	Anhydrous B_2_O_3_	56.00	4.42	56.00	4.36	56.00	4.35
MgO	Magnesium Oxide MgO (Bac)	112.00	8.84	112.00	8.72	112.00	8.70
Al_2_O_3_	T 61 Alumina, 100 mesh (Al_2_O_3_)	226.00	17.84	226.00	17.60	226.00	17.56
SiO_2_	Milled African Sand (SiO_2_)	733.00	57.86	733.00	57.09	733.00	56.95
K_2_O	K_2_CO_3_ dry (5.6 g)	3.82	0.30				
K_2_O	K_2_C_2_O_7_ (19.4 g)	6.21	0.49				
CaO	Plaster of Paris (CaSO4*1/2 H_2_O, 208 g)	80.36	6.34	80.36	6.26	80.36	6.24
TiO_2_	Titanium Dioxide TiO_2_ (F.M.A.)	10.00	0.79				
V_2_O_3_	Vandium Pentoxide A.R. (V_2_O_5_ 10 g)			8.24	0.64		
Cr_2_O_3_	K_2_C_2_O_7_ (19.4 g)	10.02	0.79				
MnO	Manganese Dioxide A.R. (MnO_2_ 10 g)			8.16	0.64		
FeO	Iron Oxide Fe_2_O_3_ (10 g)	9.00	0.71				
CoO	Cobalt Oxide, A.R. (CoO)			10.00	0.78		
NiO	Nickel Oxide NiO, A.R.					10.00	0.78
CuO	Copper Oxide, Black, A.R. (CuO)			10.00	0.78		
ZnO	Zinc Oxide ZnO (F.G.S.-8)					10.00	0.78
Rb_2_O	Rubidium Carbonate (Rb_2_CO_3_ 12.4 g)					10.04	0.78
SrO	Strontium Carbonate (Allied, 14.4 g)					10.11	0.79
Y_2_O_3_	Yttrium Oxide (Y_2_O_3_)					10.00	0.78
ZrO_2_	ZrO_2_ (Tizon)					10.00	0.78
Cs_2_O	Cesium Carbonate (Cs_2_CO_3_ 11.7 g)			10.12	0.79		
BaO	BaCO_3_ Allied 1404 (12.9 g)			10.02	0.78		
La_2_O_3_	Lanthanum Oxide (La_2_O_3_)			10.00	0.78		
Ce_2_O_3_	Cerium Oxide CeO_2_ (W.R. Grace, 10 g)	10.49	0.83				
HfO_2_	Hafnium Oxide (HfO_2_)	10.00	0.79				
PbO	Lead Oxide PbO (E.F.)					10.00	0.78
ThO_2_	Thorium Oxide (ThO_2_)			10.00	0.78		
UO_2_	Uranium Oxide U_3_O_8_ (10 g)					9.62	0.75
		
Sum		1266.9	100	1283.90	100	1287.13	100

a Gram quantities for source materials are weighed-in amounts of reagents containing carbonate or water which was lost during melting. Other oxides recalculated to commonly used oxidation state (e.g., Fe as FeO rather than Fe_2_O_3_) for purposes of reporting. No information exists as to exact oxidation state. Reagent total weights are 1400 g for 95IRV, 1419.6 g for 95IRW, and 1421.8 g for 95IRX, all prior to melting.

**Table 2:** Bulk chemical analyses of Corning glass 95IRV, concentration in mass fraction ×10^2^

Oxide	V1	V2	V3a	V3b	V3c	V4	V5	V6a	V6b	V6c	V7a	V7b
B_2_O_3_		4.44										
Na_2_O							nd	0.018	0.017			
MgO	8.73						8.79	8.93				
Al_2_O_3_	18.70						18.20	18.49				
SiO_2_	57.70						57.23	57.54				
P_2_O_5_								nd	<0.004	<0.007		
K_2_O	0.74	0.84	0.83	0.82	0.83	0.78	0.783				0.781	0.727
CaO	6.50						6.27	6.47				
TiO_2_	0.79		0.80	0.80	0.80	0.79	0.814			0.843	0.725	0.718
V_2_O_3_							nd	0.001	0.001			
Cr_2_O_3_	0.77		0.76	0.76	0.76		0.75	0.713	0.715			
MnO							int	0.006	0.006		0.005	0.004
FeO	0.75		0.70	0.71	0.71	0.74	0.764	0.805	0.806		0.749	0.703
CoO								0.001	0.001		0.001	0.001
NiO							nd	0.001	0.001		0.020	0.020
CuO								0.001	0.001		0.003	0.003
ZnO							0.008	0.006	0.006		0.001	0.001
Rb_2_O							nd					
SrO							0.075			0.077	0.096	0.072
Y_2_O_3_							0.010			0.008	0.003	0.006
ZrO_2_							0.027	0.028	0.029	0.032	0.023	0.024
Cs_2_O												
BaO							0.020			0.015	0.013	0.014
La_2_O_3_								0.002				
Ce2O_3_						0.76				0.868		
HfO_2_								0.709	0.716	0.807		
PbO								0.008	0.007		0.007	0.007
ThO_2_										0.007	0.006	0.023
UO_2_								<0.002	<0.004	<0.002		

Key to analyses: V1 Colorimetry (Mg, Al, Si, Ca, Ti, Cr, and Fe) and atomic absorption spectrophotometry (K). Analyst: Eugene Jarosewich, Smithsonian Institution V2 Gravimetry (B) and atomic absorption spectrophotometry (K). Analyst: J.L. Elize, Corning V3a,b,c Atomic absorption spectrophotometry. Analyst: Jun Ito, University of Chicago. V3a from rod 1-3 (beginning), V3b from rod 5-3 (middle), V3c from rod 9-2 (end of cane sequence) V4 X-ray fluorescence. Analyst: unknown, Johnson Space Center V5 X-ray fluorescence. Analyst: Emily Kluk, Los Alamos National Laboratory (nd, not detected, int, x-ray interference) V6a,b,c ICP-AES. Analyst: Dale Counce, Los Alamos National Laboratory, V6a,b using microwave acid digestion (samples A and B), V6c using LiBO_2_ flux dissolution V7a,b ICP-AES. Analyst: Carol Nabelek, University of Missouri, using microwave acid digestion (samples A and B)

**Table 3:** Bulk chemical analyses of Corning glass 95IRW, concentration in mass fraction ×10^2^

Oxide	W1	W2	W3a	W3b	W3c	W4	W5	W6a	W6b	W6c	W7a	W7b
B_2_O_3_		4.39										
Na_2_O							nd	<0.002	<0.005			
MgO						8.64	8.77					
Al_2_O_3_						17.98	18.26					
SiO_2_						56.78	56.58					
P_2_O_5_						0.010	nd	<0.004	<0.008			
K_2_O						0.020	nd	0.024	0.022		0.014	0.017
CaO						6.33	6.45					
TiO_2_						0.015	nd			0.005	0.006	0.006
V_2_O_3_			0.64	0.63	0.64		0.640	0.644	0.654			0.620
C_2_O_3_							int	0.002	0.002			
MnO	0.66		0.65	0.65	0.65		0.622	0.621	0.631			0.610
FeO						0.080	0.084	0.087	0.086			
CoO	0.71		0.73	0.72	0.72			0.753	0.760		0.728	0.747
NiO							0.009	0.007	0.007		0.002	0.002
CuO			0.68	0.68	0.68			0.715	0.721		0.707	0.719
ZnO							0.007	0.009	0.009		0.008	0.008
Rb_2_O							nd					
SrO							0.041			0.047	0.039	0.048
Y_2_O_3_							0.013			0.012	0.003	0.009
ZrO_2_							int	0.005	0.005	0.006	0.010	
Cs_2_O		0.72	0.72	0.72	0.70							
BaO			0.77	0.78	0.78		0.785			0.784		0.754
La_2_O_3_						0.78				0.786		
Ce_2_O_3_												
HfO_2_								0.002	0.004	0.005	0.002	0.002
PbO								0.006	0.006		0.006	0.006
ThO_2_										0.838		
UO_2_								<0.002	<0.004	<0.002	0.001	0.001

Key to analyses: W1 Colorimetry. Analyst: Eugene Jarosewich, Smithsonian Institution W2 Gravimetry (B) and atomic absorption spectrophotometry (Cs). Analyst: J.L. Elize, Corning W3a,b,c Atomic absorption spectrophotometry. Analyst: Jun Ito, University of Chicago. W3a from rod 1-3 (beginning), W3b from rod 5-3 (top), W3c from rod 9-2 (end of cane sequence) W4 X-ray fluorescence. Analyst: unknown, Johnson Space Center W5 X-ray fluorescence. Analyst: Emily Kluk, Los Alamos National Laboratory (nd, not detected, int, x-ray interference) W6a,b,c ICP-AES. Analyst: Dale Counce, Los Alamos National Laboratory, W6a,b using microwave acid digestion (samples A and B), W6c using LiBO_2_ flux dissolution W7a,b ICP-AES. Analyst: Carol Nabelek, University of Missouri, using microwave acid digestion (samples A and B)

**Table 4:** Bulk chemical analyses of Corning glass 95IRX, concentration in mass fraction ×10^2^

Oxide	X1	X2	X3a	X3b	X3c	X4	X5	X6a	X6b	X6c	X7a	X7b
B_2_O_3_												
Na_2_O							nd	<0.003	<0.005			
MgO						8.57	8.56					
Al_2_O_3_						17.94	18.13					
SiO_2_						57.01	57.44					
P_2_O_5_						nd	nd	<0.005	<0.008			
K_2_O						int	int	0.035	0.028			
CaO						6.20	6.47					
TiO_2_						0.007	nd			0.004	0.005	0.007
V_2_O_3_							0.004	0.003	0.003		0.002	0.002
C_2_O_3_							0.001	0.001	0.001			
MnO							nd	0.005	0.006		0.003	0.004
FeO						0.070	0.050	0.060	0.071			
CoO								0.004	0.004		0.004	0.004
NiO	0.70		0.74	0.72	0.71			0.747	0.732			0.764
CuO								0.005	0.005		0.005	0.006
ZnO	0.75		0.78	0.79	0.79			0.804	0.790			0.808
Rb_2_O		0.50	0.51	0.50	0.49		0.469					
SrO			0.79	0.78	0.78		0.703			0.780		0.737
Y_2_O_3_							0.886			0.816		
ZrO_2_		0.76					int	0.782	0.777	0.835		
Cs_2_O												
BaO							0.018			0.019	0.014	0.016
La_2_O_3_										0.006	0.002	0.003
Ce_2_O_3_												
HfO_2_								0.012	0.015	0.017	0.023	0.028
PbO			0.75	0.74	0.76			0.759	0.761			
ThO_2_										0.016	0.014	0.017
UO_2_								0.744	0.750	0.752	0.768	

Key to analyses: X1 Colorimetry. Analyst: Eugene Jarosewich, Smithsonian Institution X2 Gravimetry (Zr), Atomic absorption spectrophotometry (Rb). Analyst: J.L. Elize, Corning X3a,b,c Atomic absorption spectrophotometry. Analyst: Jun Ito, University of Chicago. X3a from rod 1-3 (beginning), X3b from rod 5-3 (middle), X3c from rod 9-2 (end of cane sequence) X4 X-ray fluorescence. Analyst: unknown, Johnson Space Center X5 X-ray fluorescence. Analyst: Emily Kluk, Los Alamos National Laboratory (nd, not detected, int, x-ray interference) X6a,b,c ICP-AES. Analyst: Dale Counce, Los Alamos National Laboratory, X6a,b using microwave acid digestion (samples A and B), X6c using LiBO_2_ flux dissolution X7a,b ICP-AES. Analyst: Carol Nabelek, University of Missouri, using microwave acid digestion (samples A and B)

**Table 5:** Summary of bulk chemistry and electron microprobe data for Corning glass 95IRV, concentration in mass fraction ×10^2^

Oxide	Vw	Vba	Vbsd	Vpa	Vpsd	Vpi	Vw-ba	Vs
B_2_O_3_	4.42	4.44					+0.5	
Na_2_O		0.018	0.001					
MgO	8.84	8.82	0.10				−0.3	
Al2O_3_	17.84	18.46	0.25				+3.5	
SiO_2_	57.86	57.49	0.24				−0.6	
P_2_O_5_								
K_2_O	0.79	0.792	0.073				+0.3	3.4
CaO	6.34	6.41	0.13				+1.2	
TiO_2_	0.79	0.787	0.040				−0.4	3.7
V_2_O_3_		0.001	0.000x	0.006	0.001	TiKβ int		
C_2_O_3_	0.79	0.747	0.023			CeLβ3 int	−5.5	2.8
MnO		0.005	0.001	0.005	0.001	MnKα peak, CrKβ int		
FeO	0.71	0.744	0.039				+4.7	2.8
CoO		0.001	0.000x	0.002	0.001	HfLL int, FeKα int		
NiO		0.011	0.011	0.00x		No int		
CuO		0.002	0.001	0.00x		HfLα int		
ZnO		0.004	0.003	0.006	0.003	ZnKα peak, HfLβ1 int		
Rb_2_O				(0)		SiKα limb, HfMβ int		
SrO		0.080	0.011	0.111	0.004	SrLα peak		
Y_2_O_3_		0.007	0.003	0.00x		No int		
ZrO_2_		0.027	0.003	0.033	0.004	ZrLα peak		
Cs_2_O				0.015	0.001	TiKα int		
BaO		0.016	0.003	0.014	0.001	BaLα peak, TiKα int		
La_2_O_3_		0.002		0.00x		TiKα int		
Ce_2_O_3_	0.83	0.760	0.076				−8.4	2.6
HfO_2_	0.79	0.744	0.055				−5.8	3.6
PbO		0.007	0.001	0.083	0.007	HfLγ1 int		
ThO_2_		0.012	0.010	0.00x		No int		
UO_2_				0.037	0.004	Ar K edge (flow)		

Total	100	100.39					+0.4	

Key: Vw Weighed–in values Vba Average of bulk chemistry data—use these values as working data for standard reference material 95IRV Vbsd Standard deviation 1*σ* of bulk chemistry data Vpa Average of electron microprobe analyses using 95–series glasses as primary standard (*n*=10, *Not corrected for x–ray peak overlaps*) Vpsd Standard deviation 1*σ* of electron microprobe data Vpi X–ray peak overlaps, if present, responsible for high blank values. No int, no observed interferences. peak, peak observed on electron microprobe wavelength scans. Ar K edge present adjacent to UM peak using flow x–ray counter. Vw–ba Percent difference in average bulk chemistry relative to weighed–in values. Vs Sigma ratio (1 *σ* actual standard deviation/1 *σ* standard deviation expected from counting statistics), homogeneity index measured using from 10 to 60 electron microprobe analysis points in point count grid

**Table 6:** Summary of bulk chemistry and electron microprobe data for Corning glass 95IRW, concentration in mass fraction ×10^2^

Oxide	Ww	Wba	Wbsd	Wpa	Wpsd	Wpi	Ww-ba	Ws
B_2_O_3_	4.36	4.39					+0.7	
Na_2_O		<0.003						
MgO	8.72	8.71	0.09				−0.2	
Al_2_O_3_	17.60	18.12	0.20				+3.0	
SiO_2_	57.09	56.68	0.14				−0.7	
P_2_O5		<0.005						
K_2_O	0.019	0.004	0.011	0.001		KKα peak		
CaO	6.26	6.39	0.09				+2.1	
TiO_2_		0.008	0.005	0.046	0.001	BaLα int		
V_2_O_3_	0.64	0.638	0.011			CsLβ2 int	−0.3	3.5
C_2_O_3_		0.002	0.000x	0.021	0.001	VKβ int, LaLβ2 int		
MnO	0.64	0.637	0.018				−0.5	2.1
FeO		0.084	0.003	0.034	0.001	FeKα peak, MnKβ int		
CoO	0.78	0.734	0.018				−6.0	2.9
NiO		0.005	0.003	0.007	0.001	NiKα peak, CoKβ int		
CuO	0.78	0.700	0.019			ZnKα peak,	−10.2	3.4
ZnO		0.008	0.001	0.007	0.002	ZnKα peak, CuKb int int		
Rb_2_O						SiKα limb		
SrO		0.044	0.004	0.075	0.003	SrLα peak		
Y_2_O_3_		0.009	0.005	0.003	0.003	Small YLα peak		
ZrO_2_		0.007	0.002	0.005	0.004	Small ZrLα peak		
Cs_2_O	0.79	0.710	0.012			BaLα int, LaLL int	−10.1	6.5
BaO	0.78	0.776	0.012				−0.6	3.5
La_2_O_3_	0.78	0.783	0.004			CsLβ1, Lβ4 int	+0.4	(3.3)
Ce_2_O_3_				0.064	0.002	Baβ1, Lβ4 int		
HfO_2_		0.003	0.001	0.001	0.002	CuKα int, CoKβ int		
PbO		0.006	0.000x	0.004	0.004	No int		
ThO_2_	0.78	0.838				MnKα II int	+7.4	2.8
UO_2_		0.001	0.000x	0.049	0.005	ThMβ int, ArK edge (flow)		

Total	100	100.32					+0.3	

Key: Ww Weighed-in values Wba Average of bulk chemistry data—use these values as working data for standard reference material 95IRW Wbsd Standard deviation 1*σ* of bulk chemistry data Wpa Average of electron microprobe analyses using 95-series glasses as primary standard (*n*=10, *Not corrected for x-ray peak overlaps*) Wpsd Standard deviation 1*σ* of electron microprobe data Wpi x-ray peak overlaps, if present, responsible for high blank values. No int, no observed interferences. peak, peak observed on electron microprobe wavelength scans. Ww-ba Percent difference in average bulk chemistry relative to weighed-in values. Ws Sigma ratio (1*σ* actual standard deviation/1*σ* standard deviation expected from counting statistics), homogeneity index measured using from 10 to 60 electron microprobe analysis points in point count grid

**Table 7:** Summary of bulk chemistry and electron microprobe data for Corning glass 95IRX, concentration in mass fraction ×10^2^

Oxide	Xw	Xba	Xbsd	Xpa	Xpsd	Xpi	Xw-ba	Xs
B_2_O_3_	4.35							
Na_2_O						ZnLβ1 int, YLβ3 int		
MgO	8.70	8.57	0.01				−1.6	
Al_2_O_3_	17.56	18.04	0.13				+2.7	
SiO_2_	56.95	57.23	0.30				+0.5	
P_2_O_5_		<0.006						
K_2_O		0.028	0.008	0.017	0.000x	KKα peak, UMβ tail		
CaO	6.24	6.34	0.19				+1.5	
TiO_2_		0.006	0.002	0.028	0.002	No int		
V_2_O_3_		0.003	0.001	0.003	0.001	No int		
C_2_O_3_		0.001	0.000x	0.001	0.001	No int		
MnO		0.005	0.001	0.004	0.001	MnKα peak		
FeO		0.063	0.010	0.030	0.001	FeKα peak		
CoO		0.004	0.000x	0.003	0.001	No int		
NiO	0.78	0.730	0.022				−6.4	4.4
CuO		0.005	0.001	0.002	0.001	CuKα peak, NiK		
ZnO	0.78	0.787	0.019			NiKβ int	+1.0	3.8
Rb_2_O	0.78	0.494	0.016			SiKα limb	−36.7	5.2
SrO	0.79	0.762	0.034			RbLβ 4 int	−3.6	3.3
Y_2_O_3_	0.78	0.851	0.049				+9.1	2.8
ZrO_2_	0.78	0.789	0.032				+1.1	3.1
Cs_2_O				0.003	0.001	No int		
BaO		0.017	0.002	0.017	0.001	BaLα peak		
La_2_O_3_		0.004	0.002	0.000x	0.000x	No int		
Ce_2_O_3_				0.009	0.002	ZnKβ 1,3 II int		
HfO_2_		0.019	0.006	0.018	0.003	ZrKα 1 II int		
PbO	0.78	0.754	0.009				−3.3	4.6
ThO_2_		0.016	0.002	0.000x	0.001	No int		
UO_2_	0.75	0.754	0.010			ArK edge (flow)	+0.5	3.1

Total	100	100.60	0.010			ArK edge (flow)	+0.5	3.1

Key: Xw Weighed–in values Xba Average of bulk chemistry data—use these values as working data for standard reference material 95IRX Xbsd Standard deviation 1*σ* of bulk chemistry data Xpa Average of electron microprobe analyses using 95–series glasses as primary standard (*n*=10, *Not corrected for x–ray peak overlaps*) Xpsd Standard deviation 1*σ* of electron microprobe data Xpi x–ray peak overlaps, if present, responsible for high blank values. No int, no observed interferences. peak, peak observed on electron microprobe wavelength scans. Xw–ba Percent difference in average bulk chemistry relative to weighed–in values. Xs Sigma ratio (1*σ* actual standard deviation/1*σ* standard deviation expected from counting statistics), homogeneity index measured using from 10 to 60 electron microprobe analysis points in point count grid

## Footnotes

1 NIST disclaimer: Certain commercial equipment, instruments, or materials are identified in this paper to foster understanding. Such identification does not imply recommendation or endorsement by the National Institute of Standards and Technology, nor does it imply that the materials or equipment are necessarily the best available for the purpose.

## References

[1] Carpenter PK (1999). Status Report on Corning Standard Glasses 95IRV, 95IRW, and 95IRX. Microsc Microanal.

[2] Carpenter P, Counce D, Kluk E, Nabelek C (2000). Characterization of Corning Standard Glasses 95IRV, 95IRW, and 95IRX, by ICP-AES, XRF, and EPMA.

[3] Govindaraju K (1994). Compilation of Working Values and Sample Description for 383 Geostandards. Geostand Newslett.

[b4-j76car] 4To obtain these standards contact Edward Vicenzi, Department of Mineral Sciences, Smithsonian Institution, Washington, DC 20560-0119, email vicenzi@volcano.si.edu, phone 202-357-2594. (Contact Paul Carpenter if disc format is necessary).

